# Development and validation of a nomogram to predict the survival and estimate surgical benefits for gastric cancer with liver metastasis receiving primary tumor resection

**DOI:** 10.3389/fonc.2024.1418548

**Published:** 2024-11-08

**Authors:** Rishun Su, Xuezeng Sun, Songyao Chen, Guofei Deng, Songcheng Yin, Yulong He, Tengfei Hao, Liang Gu, Changhua Zhang

**Affiliations:** ^1^ Digestive Diseases Center, The Seventh Affiliated Hospital of Sun Yat-sen University, Shenzhen, China; ^2^ Guangdong Provincial Key Laboratory of Digestive Cancer Research, The Seventh Affiliated Hospital of Sun Yat-sen University, Shenzhen, China; ^3^ Department of Gastrointestinal Surgery, The First Affiliated Hospital of Sun Yat-Sen University, Guangzhou, China

**Keywords:** gastric cancer, liver metastasis, prognosis, surgery, nomogram

## Abstract

**Background:**

Surgical treatment has been widely controversial for gastric cancer accompanied by liver metastasis (GCLM). This paper aims to develop and validate a nomogram to predict the survival and estimate surgical benefits for GCLM patients.

**Methods:**

A total of 616 GCLM patients from the Surveillance, Epidemiology, and End Results Program (SEER) database and 74 GCLM patients receiving primary tumor resection (PTR) from the Chinese center were included in this study. Patients from the SEER database were divided into training set (with PTR) (n=493) and non-operative set (without PTR) (n=123). Patients undergoing PTR from China were included as external validation set. Independent risk factors associated with the overall survival of GCLM patients undergoing PTR were identified in the training set via log-rank test and Cox regression analysis. Afterwards, a comprehensive model and corresponding nomogram were constructed and validated by validation set.

**Results:**

The survival of patients undergoing PTR (n=493) was longer than that without PTR (n=123) (log-rank test, *p*<0.0001) in SEER cohort. T stage (HR=1.40, 95% CI=1.14, 1.73), differentiation grade (HR=1.47, 95% CI=1.17, 1.85), non-hepatic metastases (HR=1.69, 95% CI=1.29, 2.21), and adjuvant therapy (HR=0.34, 95% CI= 0.28, 0.42) were closely related with the survival of GCLM with PTR, and thus, a four-factor nomogram was established. However, GCLM patients receiving PTR in the high-risk subgroup (n=255) screened out by the nomogram did not have better survival outcomes compared with patients without PTR (n=123) (log-rank test, *p*=0.25).

**Conclusions:**

The nomogram could predict survival of GCLM patients receiving PTR with acceptable accuracy. In addition, although PTR did improve the survival of whole GCLM patients, patients in the high-risk subgroup were unable to benefit from PTR, which could assist clinicians to make decisions for the treatment of GCLM.

## 1 Introduction

Gastric cancer is one of the most commonly seen gastrointestinal malignancy and the fourth leading cause of cancer-related deaths in the world (1, 2). Liver is the most common target organ for the metastasis of gastric cancer. Roughly 3%–14% of gastric cancer patients are diagnosed as gastric cancer with liver metastasis (GCLM) at their first visit, and the occurrence of liver metastasis greatly affects the survival of patients. For patients with GCLM, especially for synchronous liver metastasis that are diagnosed preoperatively, there are still more divergences on its treatment. Current treatment protocols in western centers do not recommend patients with distant metastases and peritoneal disease to receive surgical treatment. Patients with hepatic metastases are traditionally treated with palliative chemotherapy (2, 3). In recent years, surgical treatment is convinced to be an effective approach to improve survival of GCLM patients, especially for patients with limited liver metastasis (4–6). However, the benefits of surgery vary greatly from person to person, in view of the significant heterogeneity among patients or among tumors and various therapies they received (5, 7). The AJCC-TNM staging system only roughly divides patients into stage IV and is unable to predict the survival and to evaluate surgical benefits of GCLM patients. There is still a lack of effective systematic prediction tools at present.

In addition, thanks to a relatively low incidence of GCLM in gastric cancer patients and low excisional rate, prognostic analysis of patients undergoing surgery has long been impaired by the small quantities of sample. A few efforts involving small groups of patients have been made to identify clinicopathological characteristics that predict survival (8, 9).

Accordingly, in this paper, we estimated surgical benefits and developed a nomogram to predict the survival for GCLM patient receiving primary tumor resection (PTR), by the use of The Surveillance, Epidemiology, and End Results (SEER) database, and validated the nomogram by the use of clinical data of patients from the First Affiliated Hospital of Sun Yat-sen University.

## 2 Materials and methods

### 2.1 Study cohorts and criteria of inclusion and exclusion

**Figure 1:**
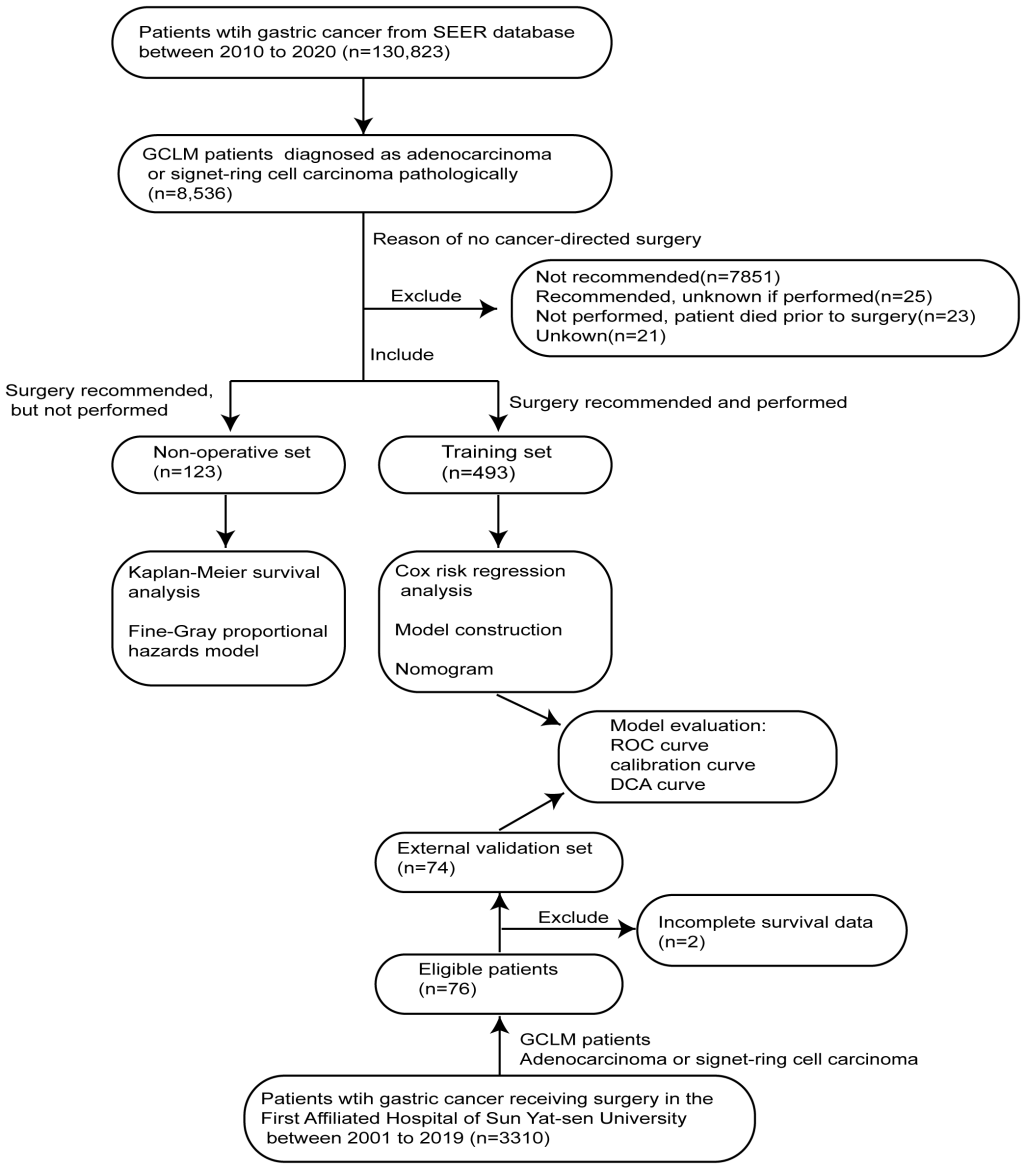
Flow-process diagram of patients screening and model construction.

The SEER*Stat software (version 8.4.1) was used to obtain clinicopathological and survival data of GCLM patients for non-operative set (without PTR) and training set (with PTR). According to the description of the SEER database, PTR indicates a surgical procedure that removes and/or destroys tissue of the primary site performed as part of the initial workup or first course of therapy. Data of all gastric cancer patients registered in the SEER database from 2010 to 2020 were downloaded by the software. For the validation set, 74 GCLM patients undergoing PTR were retrospectively screened out from 3,310 gastric cancer patients who underwent surgical treatment in the First Affiliated Hospital of Sun Yat-sen University at Guangzhou, Guangdong Province, China from 2001 to 2019. The strategy for screening patients is shown in **Figure 1**.

Specifically, the inclusion criteria of training and validation sets are as follows: (1) patients who were diagnosed as gastric adenocarcinoma (AYA site recode 2020 Revision, including signet ring cell carcinoma) pathologically, (2) patients confirmed with hepatic metastasis clearly (SEER Combined Mets at DX-liver), and (3) patients who were recommended and performed for PTR. The exclusion criteria for both non-operative and training sets were as follows: (1) patients lacking survival data, (2) patients with other primary malignant tumors, and (3) those whose tumors have uncertain pathological type. In addition, the inclusion criteria of the non-operative set include the following: (1) patients who were diagnosed as gastric adenocarcinoma (AYA site recode 2020 Revision, including signet ring cell carcinoma) pathologically, (2) patients confirmed with hepatic metastasis clearly (SEER Combined Mets at DX-liver), and (3) patients who were recommended for PTR but not performed (reason: no cancer-directed surgery).

### 2.2 Clinical outcomes and variables

The overall survival (OS) was defined as the time interval from cohort entry to death due to any cause. Cancer-specific survival (CSS) was defined as the time interval from cohort entry to death due to gastric cancer, whereas non-cancer-specific survival (Non-CSS) was defined as the time interval from entry to death that resulted from non-cancer diseases.

For patients in non-operative and training sets, information of OS (months), survival status (death or alive), and cause of death were extracted from the SEER database. Death causes were identified according to SEER cause-specific death classification: Dead (attributable to this cancer dx), Alive or dead of other cause, and Dead (missing/unknown COD). For the validation set, all eligible GCLM patients were followed from the date of a definite diagnosis until the date of death or 31 December 2019, whichever came first.

The variables of all GCLM patients who meet the above criteria were collected as follows: (1) demographic data, including age at diagnosis (<60/≥60), sex (male/female), and race (white/black/Asian or pacific islander/other); (2) characteristics of tumors, including tumor site (distal stomach: gastric antrum and pylorus/proximal stomach: gastric body and cardia and cundus/other: greater curvature and lesser curvature and overlapping lesion and stomach, NOS), histological type (adenocarcinoma/signet ring cell) tumor grade(I/II/III/IV/Unknown), T stage (T1/T2/T3/T4/TX), N stage (N0/N1/N2/N3/NX), non-hepatic metastases (lung, bone, brain, distant lymph nodes metastases, peritoneal metastasis, and so on); (3) perioperative adjuvant therapy, including radiotherapy and chemotherapy; and (4) survival data, including OS (months), survival status (death or alive), and causes of death.

### 2.3 Ethics statement

The study was conducted in accordance with the Declaration of Helsinki, and the protocol was approved by the Ethics Committee of the First Affiliated Hospital of Sun Yat-sen University (No. KY-2020-024-01). Informed consent was waived by the committee, since this is a retrospective study and there were no biological specimens of patients that were used in the research. The ethical review for data obtained from SEER database was not required.

### 2.4 Statistical analysis

R (4.3.0) software and GraphPad Prism 8.0 were used for statistical analysis and visualization of results. Continuous data were presented as mean ± standard deviation (SD) or median (interquartile range, *IQR*), while discrete data were presented as frequency and percentage (%).

For univariate analysis, Mann–Whitney U-test and Student’s t-test were used to compare the difference in continuous data between different groups, while chi-square test was used for the discrete data. In addition, Kaplan–Meier method, log-rank test, and Fine–Gray proportional hazards model were used to compare OS and CSS. Univariate Cox risk regression analysis was employed to filter out potential predictive factors preliminarily. For multivariate analysis, multivariate Cox risk regression analysis and step-wise regression analysis were used to identify significant prognostic factors for the construction of predictive model and nomogram. Hazard ratios (HRs) and 95% confidence intervals (95% CIs) of predictive factors were calculated. Furthermore, to evaluate the prognostic power of the model and nomogram, via time-dependent ROC curve analysis for the 1-, 3-, and 5-year survival rates, calibration curve and decision curve analysis (DCA) were performed by the use of R packages “timeROC,” “rms,” “ggDCA,” and “ggplot2.” The above statistical tests are bilateral tests. *p*<0.05 was considered statistically significant.

### 2.5 Development and validation of the nomogram

In the training set, variables with statistical significance of univariate Cox regression analysis were included in the multivariate Cox regression analysis with stepwise regression. Afterwards, variables with a *p*-value <0.05 in multivariate Cox risk regression analysis were integrated to construct proportional hazard regression model to predict the survival risk. A predictive model was built, and the risk score of each patient in the training cohort was calculated to determine the predictive role of this model. R package “rms” was used to draw a nomogram corresponding to the predictive model. The prognostic power of the model and nomogram were evaluated by time-dependent ROC curve analysis, calibration curve, and DCA. To validate the nomogram, we calculated the risk score of each patient in the external validation cohort by the use of the predictive model and then investigated the predictive significance of the model. Equally, the performance of this model was assessed using time-dependent ROC curve analysis and DCA.

### 2.6 Risk groups

In the training set, according to the median of risk score computed by the predictive model, we divided patients into high- and low-risk groups. Patients in the high-risk group had a risk score greater than or equal to the median, whereas patients with a risk score below the median would be included in the low-risk group. In the validation set, employing the same cutoff point of the training set (the median score), patients were also divided into high- and low-risk groups.

## 3 Results

### 3.1 Demographic and clinicopathological characteristics of three cohorts

In total, 616 patients diagnosed with GCLM between 2010 and 2020 from SEER database were screened out from 130,823 gastric cancer patients in our study. There were 421 men (68.3%) and 195 women (31.7%), and the patients mainly concentrated in the age group of over 60 years old. Specifically, 123 GCLM patients in whom PTR was suggested to be performed but not receiving surgical treatment ultimately were enrolled in the non-operative set, while 493 patients receiving PTR treatment were enrolled in the training set. At the same time, 74 GCLM patients receiving PTR from the First Affiliated Hospital of Sun Yat-sen University were identified from 3,310 gastric cancer patients as the validation set (**Figure 1**). The description and comparison of demographic and clinicopathological features of these three cohorts are shown in **Table 1**.

**Table 1:** Demographic and clinicopathological characteristics of non-operative set, training set, and validation set.

Variables		Non-operative set (n=123)	Training set (n=493)	Validation set (n=74)	*p*_1_^*^	*p*_2_^*^
Age at diagnosis (years)	<60	23 (18.7%)	130 (26.4%)	15 (20.3%)	0.100	0.328
	≥60	100 (81.3%)	363 (73.6%)	59 (79.7%)		
Sex	Female	37 (30.1%)	158 (32%)	22 (29.7%)	0.756	0.790
	Male	86 (69.9%)	335 (68%)	52 (70.3%)		
Race	White	88 (71.5%)	303 (61.5%)	0 (0%)	0.001	<0.001
	Black	10 (8.1%)	101 (20.5%)	0 (0%)		
	Asian or Pacific Islander	20 (16.3%)	84 (17%)	74 (100%)		
	Other	5 (4.1%)	5 (1%)	0 (0%)		
Histological type	Adenocarcinoma	113 (91.9%)	457 (92.7%)	69 (93.2%)	0.904	1.000
	Signet ring	10 (8.1%)	36 (7.3%)	5 (6.8%)		
Primary tumor site	Distal stomach^2^	17 (13.8%)	174 (35.3%)	25 (33.8%)	<0.001	0.869
	Proximal stomach^2^	65 (52.8%)	158 (32%)	26 (35.1%)		
	Other^2^	41 (33.3%)	161 (32.7%)	23 (31.1%)		
Grade	Grade I&II	32 (26%)	130 (26.4%)	22 (29.7%)	0.015	0.002
	Grade III&IV	50 (40.7%)	257 (52.1%)	49 (66.2%)		
	Unknown	41 (33.3%)	106 (21.5%)	3 (4.1%)		
N stage	N0&1	67 (54.5%)	220 (44.6%)	36 (48.6%)	<0.001	0.113
	N2&3	8 (6.5%)	246 (49.9%)	30 (40.5%)		
	NX	48 (39%)	27 (5.5%)	8 (10.8%)		
T stage	T2&3	47 (38.2%)	223 (45.2%)	33 (44.6%)	<0.001	0.552
	T4	14 (11.4%)	226 (45.8%)	37 (50%)		
	TX	62 (50.4%)	44 (8.9%)	4 (5.4%)		
Radiotherapy	Yes	12 (9.8%)	64 (13%)	–	0.412	–
	No/Unknown	111 (90.2%)	429 (87%)	–		
Chemotherapy	Yes	37 (30.1%)	278 (56.4%)	39 (52.7%)	<0.001	0.180
	No/Unknown	86 (69.9%)	215 (43.6%)	35 (47.3%)		
Adjuvant therapy	Yes	38 (30.9%)	279 (56.6%)	39 (52.7%)	<0.001	0.169
	No/Unknown	85(69.1%)	214 (43.4%)	35 (47.3%)		
Bone metastasis	Yes	13 (10.6%)	20 (4.1%)	1 (1.4%)	<0.001	0.110
	No	97 (78.9%)	454 (92.1%)	73 (98.6%)		
	Unknown	13 (10.6%)	19 (3.9%)	0 (0%)		
Brain metastasis	Yes	1 (0.8%)	7 (1.4%)	0 (0%)	<0.001	0.179
	No	107 (87%)	471 (95.5%)	74 (100%)		
	Unknown	15 (12.2%)	15 (3%)	0 (0%)		
Lung metastasis	Yes	21 (17.1%)	36 (7.3%)	3 (4.1%)	<0.001	0.241
	No	87 (70.7%)	446 (90.5%)	71 (95.9%)		
	Unknown	15 (12.2%)	11 (2.2%)	0 (0%)		
Distant lymph nodes metastasis	Yes	12 (9.8%)	24 (4.9%)	0 (0%)	0.034	<0.001
	No	21 (17.1%)	123 (24.9%)	74 (100%)		
	Unknown	90 (73.2%)	346 (70.2%)	0 (0%)		
Peritoneal metastasis	Yes	–	–	21(28.4%)	–	–
	No	–	–	53(71.6%)		
Other metastasis^1^	Yes	8 (6.5%)	36 (7.3%)	–	0.917	–
	No	27 (22%)	113 (22.9%)	–		
	Unknown	88 (71.5%)	344 (69.8%)	–		
Non-hepatic metastasis	Yes	37 (30.1%)	92 (18.7%)	27 (36.5%)	0.008	<0.001
	No/Unknown	86 (69.9%)	401 (81.3%)	47 (63.5%)		
Overall survival (months)	Median (*IQR*)	3 (1, 7)	8 (3, 18)	9.5 (4, 19.25)	<0.001	0.188
Survival status	Alive	11 (8.9%)	68 (13.8%)	22 (29.7%)	0.198	<0.001
	Dead	112 (91.1%)	425 (86.2%)	52 (70.3%)		
Cause of death	Death due to cancer	98(87.5%)	383(90.1%)	–	0.375	–
	Death due to other cause	14(12.5%)	42(9.9%)	–		
Cancer-specific survival (months)	Median (*IQR*)	2(1,6)	6(2,13)	–	<0.001	–

*p_1_: Non-operative set vs. training set; *p*_2_: validation set vs. training set.

^1^Other metastasis: distant metastasis in known site(s) other than bone, brain, liver, lung, distant lymph nodes, or generalized metastases.

^2^Distal stomach: gastric antrum and pylorus;

•proximal stomach: gastric body, cardia, and cundus; other: greater curvature, lesser curvature; overlapping lesion, and stomach.

There were no missing survival data for the three cohorts because these patients have been excluded. Patients in two SEER cohorts have lost the information about peritoneal metastasis (n=616), while patients in the external validation set have lost several predictors, including cause of death (n=74) and radiotherapy (n=74). Three groups of patients have similar demographic features, including age and sex. There are more white than other races in the two SEER cohorts (71.5% in the non-operative set; 61.5% in the training set), while all patients in the external validation set were Asian. The median OS and CSS were 3.0 (*IQR*=1.0, 7.0) months and 2.0 (*IQR*=1.0, 6.0) months, respectively, in the non-operative set, while they were 8.0 (*IQR*=3.0, 18.0) months and 6.0 (*IQR*=2.0, 13.0) months, respectively, in the training set. Meanwhile, in the validation set, median overall survival time was 9.5 (*IQR*=4.0, 19.25) months, which was similar to that in the training set (Mann–Whitney U-test, *p* > 0.05). Additionally, patients of the non-operative set had a higher incidence of non-hepatic metastasis (30.1% vs. 18.7%) and lower rate of adjuvant therapy (30.9% vs. 56.6%) in contrast to the training set (with PTR).

### 3.2 Patients in the SEER cohort can benefit from PTR

**Figure 2:**
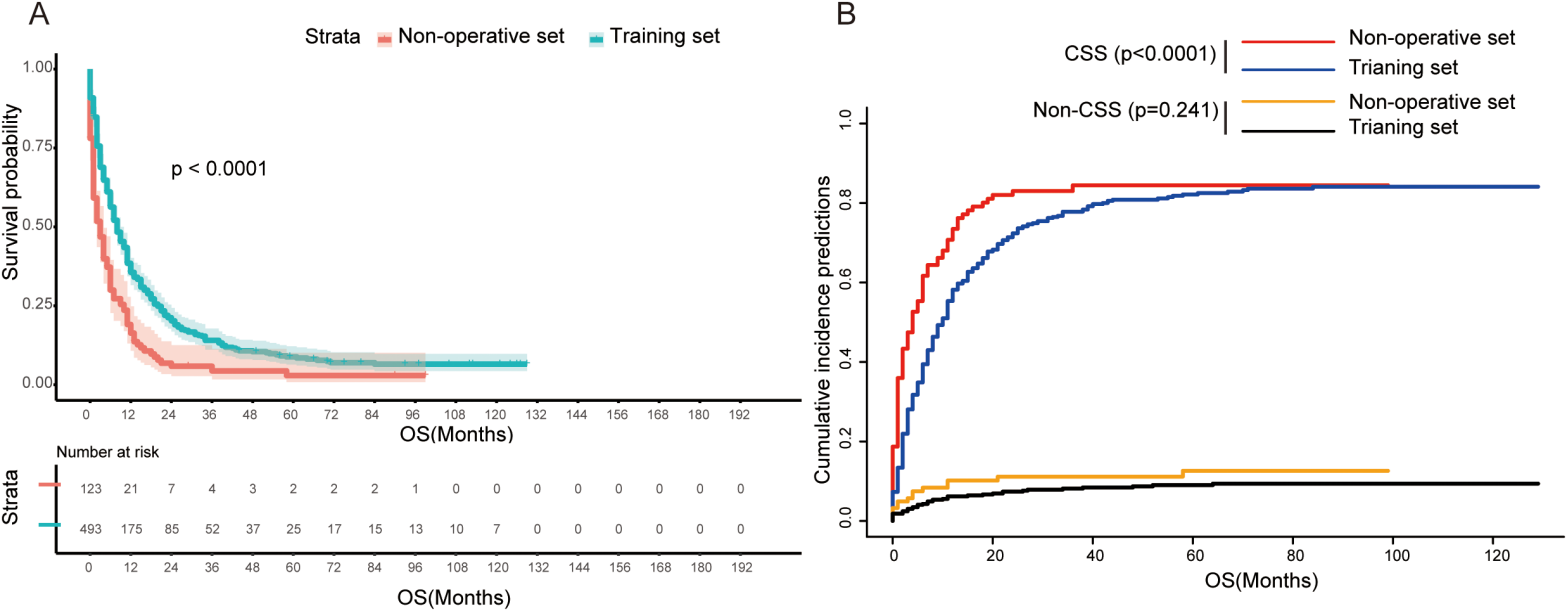
**(A)** Kaplan–Meier survival curve between non-operative set and training set (log-rank test, *p*<0.0001). **(B)** Competing risk of cancer-specific death (Fine–Gray test, *p*<0.0001) and non-cancer-specific death (Fine–Gray test, *p*=0.241). OS, overall survival; CSS, cancer-specific survival.

Patients in non-operative and training sets both were recommended for PTR treatment in the SEER cohort. Our result showed that patients in the training set who underwent PTR had a better prognosis in contrast to patients in the non-operative set—8 months versus 3 months in median OS and 6 months versus 2 months in median CSS (**Table 1**). The result of Kaplan–Meier analysis further confirmed this difference statistically (log-rank *p* < 0.001, **Figure 2A**). Furthermore, we employed a single-factor competing risk model to minimize bias caused by death not related to cancer. The Fine–Gray model showed that after controlling for competitive risk events (i.e., dead due to other causes), there was a statistical correlation between the cumulative death risk and PTR treatment (Fine–Gray test, *p <*0.001, **Figure 2B**). Furthermore, to eliminate the potential confounding effect brought by a longer time duration, we carried out a subgroup analysis of valid data of the SEER from 2010 to 2020. The result indicated that surgical treatment still benefited GCLM patients across subgroups with different time spans (“2010 to 2014” vs. “2015 to 2020”) (**Supplementary Figure S1**). In addition, the subgroup analysis stratified by T stage, N stage, non-hepatic metastases, and differentiation grade was implemented to distinguish the more detailed groups of GCLM who can benefit more from PTR. As shown in **Supplementary Figure S2**, the four variables can further predict the prognosis for GCLM patients in PTR subgroup. GCLMs with preferable clinicopathological characteristics (T1&2, N0&1, Grade I&II, and non-hepatic metastases free) are more likely to benefit from PTR and have a much better survival compared with non-surgical patients.

### 3.3 Variables affecting the survival of GCLM patients with PTR

**Figure 3:**
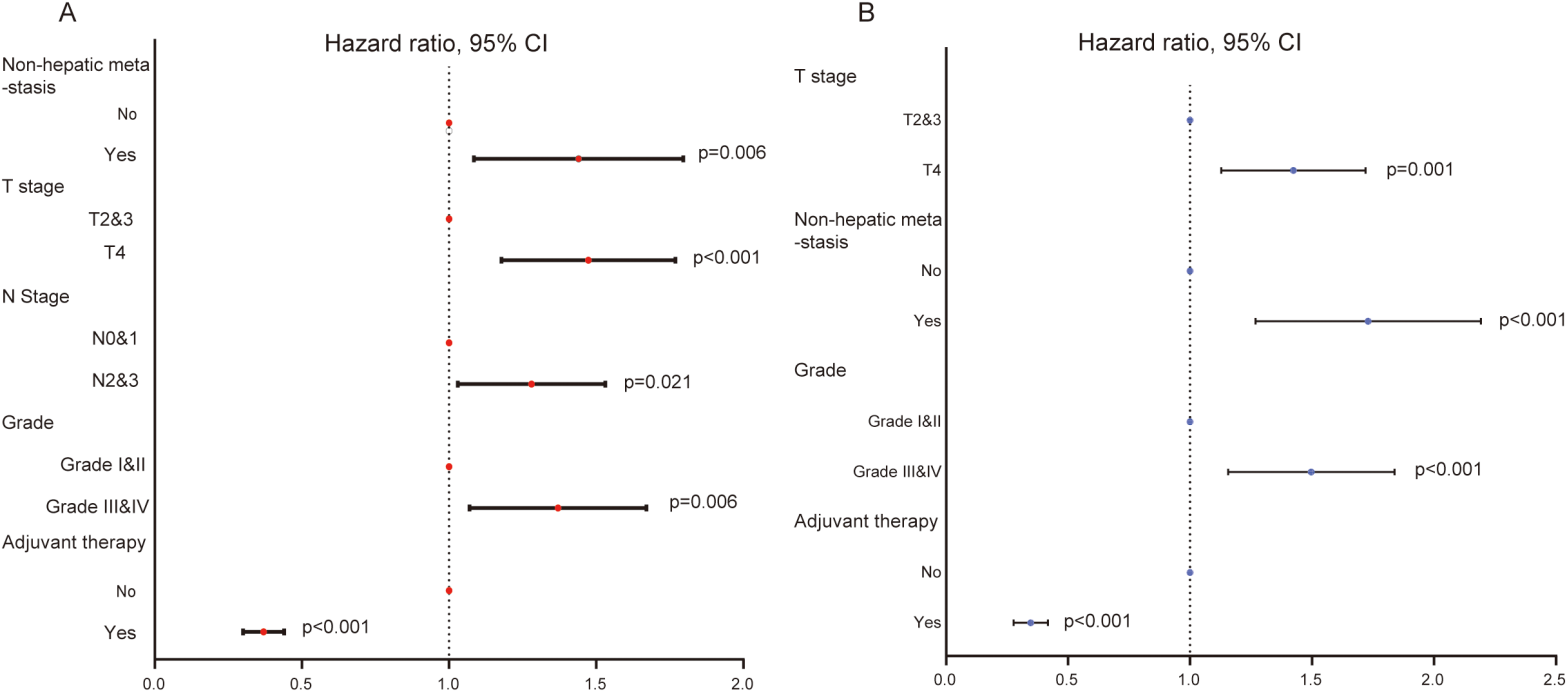
**(A)** The forest plot of univariate Cox regression analysis showing five variables affecting the OS for GCLM patients. **(B)** The forest plot of multivariate Cox regression analysis identifying four variables as independent factors of OS for GCLM patients.

Given the important role of PTR surgery for the prognosis of GCLM patients, we ulteriorly identified significant factors that were related to overall survival of patients in the training set by univariate and multivariate Cox regression analyses. A total of 10 variables were submitted for preliminary analysis, and the results are summarized in **Table 2**. In particular, the result of univariate Cox regression showed that five variables, including T stage, N stage, differentiation grade, non-hepatic metastases, and adjuvant therapy, were significantly related with the survival of CGLM patients with PTR (**Table 2**, **Figure 3A**). Subsequently, these candidate factors were included in multivariate Cox regression analysis to determine the independent risk factors. As depicted by the forest plot, T stage (p=0.001, HR=1.40, 95% CI=1.14, 1.73), grade (p<0.001, HR=1.47, 95% CI=1.17, 1.85), non-hepatic metastases (p<0.001, HR=1.69, 95% CI=1.29, 2.21), and adjuvant therapy (p<0.001, HR=0.34, 95% CI= 0.28,0.42) were independent factors that influence overall survival of GCLM patients (**Table 2**, **Figure 3B**).

**Table 2:** Results of univariate and multivariate Cox regression analyses.

	Univariate Cox regression			Multivariate Cox regression		
Variables	HR^1^	95% CI^1^	*p*-value	HR^1^	95% CI^1^	*p*-value
Age						
<60	1(reference)					
≥60	1.19	0.96, 1.49	0.12			
Sex						
Female	1(reference)					
Male	1.19	0.97, 1.46	0.10			
Race						
Asian/Pacific Islander	1(reference)					
Black	0.78	0.57, 1.06	0.11			
Other & Unknown	0.51	0.16, 1.61	0.3			
White	0.95	0.73, 1.23	0.7			
Histological type						
Adenocarcinoma	1(reference)					
Signet ring	1.12	0.79, 1.59	0.5			
Primary site						
Distal stomach	1(reference)					
Other	1.07	0.85, 1.35	0.6			
Proximal stomach	0.81	0.64, 1.03	0.082			
T stage						
T2&3	1(reference)			1(reference)		
T4	1.45	1.19, 1.78	<0.001	1.40	1.14, 1.73	0.001
TX	1.04	0.73, 1.48	0.8	0.98	0.65, 1.47	>0.9
N stage						
N0&1	1(reference)			1(reference)		
N2&3	1.26	1.04, 1.54	0.021	1.08	0.88, 1.33	0.5
NX	1.36	0.87, 2.11	0.2	1.64	1.00, 2.68	0.05
Non-hepatic metastasis						
No/Unknown	1(reference)			1(reference)		
Yes	1.41	1.10, 1.81	0.006	1.69	1.29, 2.21	<0.001
Differentiation grade						
Grade I&II	1(reference)			1(reference)		
Grade III&IV	1.35	1.08, 1.68	0.008	1.47	1.17, 1.85	<0.001
Unknown	0.90	0.67, 1.21	0.5	0.98	0.71, 1.35	0.9
Adjuvant therapy						
No	1(reference)					
Yes	0.37	0.30, 0.44	<0.001	0.34	0.28, 0.42	<0.001

^1^HR, hazard ratio; CI, confidence interval.

### 3.4 The construction of the predictive model and nomogram

Eventually, four variables, T stage, differentiation grade, non-hepatic metastasis, and adjuvant therapy, were used for the construction of predictive model and nomogram in the training set. The formula to calculate risk score was as follows:


$$\begin{array}{l} \text{Risk score}=\text{h}0(t)*\text{exp}[\text{T Stage}(\text{T}2\&3=1,\text{T}4=1.4)+\text{non}\\ -\text{hepatic metastasis}(\text{no}=1,\text{yes}=1.69)\\ +\text{Differentiation grade}(\text{GradeI}\&\text{II}=1,\text{Grade III}\&\text{IV}=1.47)\\ +\text{Adjuvant therapy}(\text{no}=1,\text{yes}=0.34)] \end{array}$$

**Figure 4:**
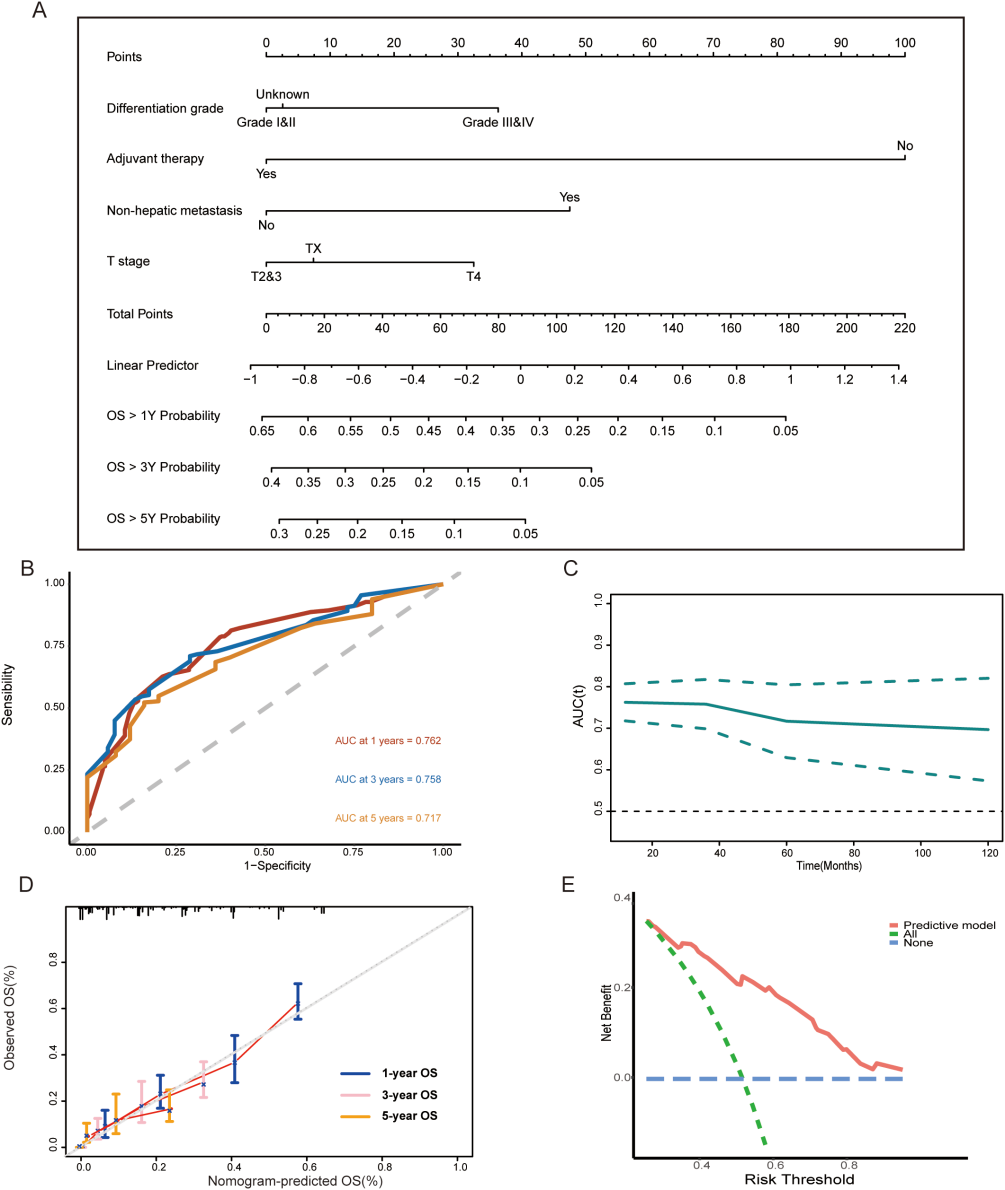
**(A)** Nomogram consisting of T stage, differentiation grade, non-hepatic metastases, adjuvant therapy, and predicting the 1-, 3-, and 5-year overall survival rates for surgical GCLM patients. **(B, C)** Time-dependent ROC curves and time-dependent AUC curve evaluating the nomogram. **(D)** Calibration curves of the nomogram for predicting of 1-, 3-, and 5-year overall survival rates. **(E)** DCA curve of the nomogram. OS, overall survival; ROC, receiver operating characteristic; AUC, the area under the curve; DCA, decision curve analysis.

Based on these results, we further established a nomogram consisting of the above four variables to predict 1-, 3-, and 5-year survival probability for GCLM patients (**Figure 4A**). Next, we performed a time-dependent ROC curve analysis to evaluate the discrimination of the model and corresponding nomogram (**Figures 4B, C**). The areas under the curve (AUCs) of the 1-, 3-, and 5-year ROC curves based on the risk score were 0.762, 0.758, and 0.717, respectively, which implied that the model had a good discriminative ability in survival prediction (**Figure 4B**). Meanwhile, as shown in **Figure 4C**, the discriminative ability of this model decreased to a stable level after 5 years, and the all AUCs at each predictive time point exceeded 0.7, which further confirmed the robust ability of the survival prediction of the model. Additionally, we also depicted the nomogram calibration plots to the consistency between our proposed nomogram and an ideal model, and the result showed that the model was close to the ideal state (**Figure 4D**). The DCA curve demonstrated that this predictive model had a pleasant clinical practicality in the training set (**Figure 4E**).

### 3.5 The estimation of the surgical benefits of patients in high-risk group

**Figure 5:**
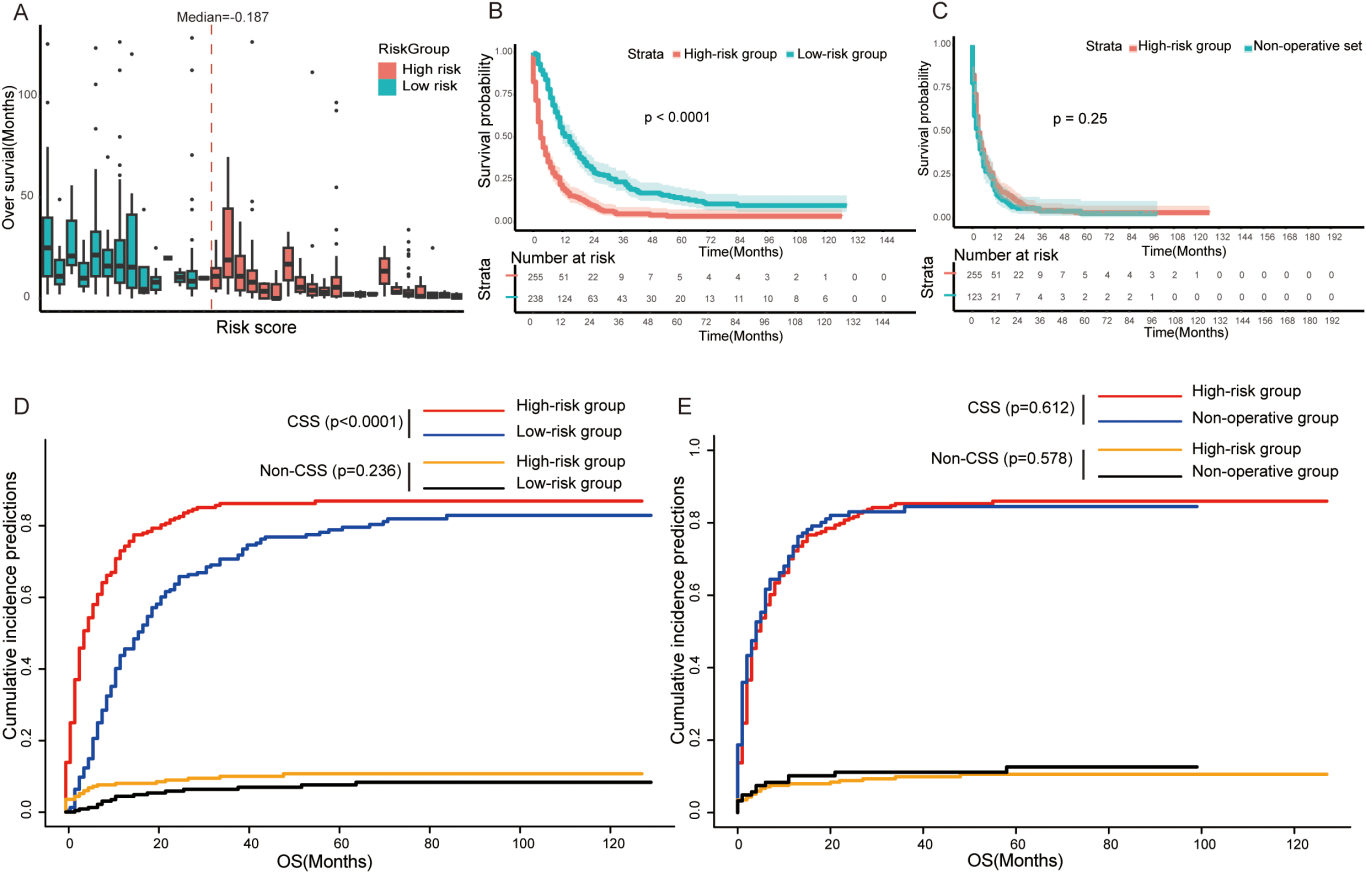
**(A)** Box plots representing the risk score distribution and overall survival of each GCLM patients. **(B, D)** Kaplan–Meier survival curve **(B)** and competing risk model **(D)** between the high-risk group and low-risk group in training set. **(C, E)** Kaplan–Meier survival curve **(C)** and competing risk model **(E)** between high-risk group and non-operative set. OS, overall survival; CSS, cancer-specific survival.

According to the median of risk score derived from the above predictive model, we divided patients into high-risk (risk score ≥ −0.1874808) and low-risk group (risk score < −0.1874808) (**Figure 5A**). The result of the survival analysis showed that the overall survival of patients in the low-risk group (n=238) (median OS=12.0 months, *IQR* =7.0, 24.8) was longer than that in the high-risk group (n=255) (median OS=3.0 months, *IQR* =1.0, 10.0) (log-rank test, p<0.0001) (**Figure 5B**). Meanwhile, patients in the high-risk group had a high risk of cancer-specific death and worse CSS (Fine–Gray test, *p* < 0.0001, **Figure 5D**). However, patients in the high-risk group who underwent surgical operation did not have a longer overall survival compared to non-surgical patients (log-rank test, *p*=0.25, **Figure 5C**). Similarly, there was no significant difference in CSS and accumulation risk of cancer-specific death between the two groups of patients (Fine–Gray test, *p* = 0.612, **Figure 5E**).

### 3.6 The external validation of the nomogram

**Figure 6:**
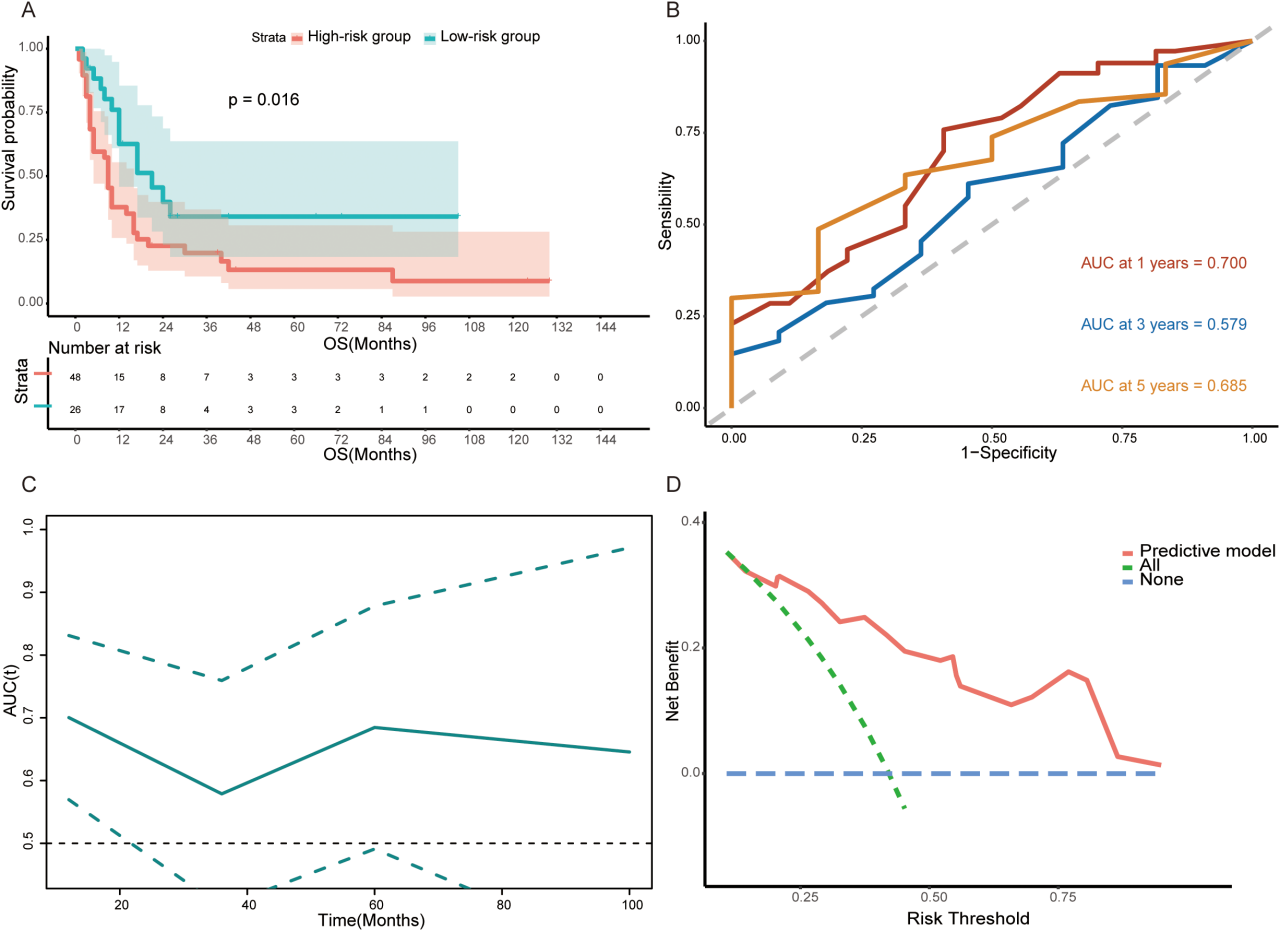
**(A)** Kaplan–Meier survival curve between the high-risk group and low-risk group in validation set (log-rank test, *p*<0.0001). **(B, C)** Time-dependent ROC curves and time-dependent AUC curve evaluating the predictive power of the nomogram in validation set. **(D)** DCA curve of the nomogram in validation set.

To further verify the robustness of the model and nomogram, we recruited 74 surgical GCLM patients from the First Affiliated Hospital of Sun Yat-sen University as the validation set. In particular, the predictive model was used to calculate the risk score of each patient in the validation set and the same threshold, i.e., the median (−0.1874808) of the risk score in the training set, was employed to define high- and low-risk subgroups. The further analysis result of the validation set is similar to that of the training set. That is, patients who underwent PTR in the low-risk group (n=26) (median OS=9.0 months, *IQR* = 4.0, 16.3) tended to have a longer overall survival time than the high-risk group (n=48) (median OS=12.0 months, *IQR* =8.0, 26.0) (log-rank test, p=0.016, **Figure 6A**). Additionally, the ROCs of 1-, 3-, and 5-year survival rates and corresponding AUCs (0.700, 0.579, and 0.685), t-AUC curve, and the DCA curve further confirmed the prognostic predictive ability of this model (**Figures 6B–D**).

## 4 Discussion

According to the latest statistics of global burden of gastric cancer, a total of 1,089,000 patients were newly diagnosed with gastric cancer. It is estimated that the number of new cases will rise to 1.77 million by 2040, and 769,000 patients died from it directly in 2020 (1, 10). Diffusion and metastasis are the important causes of cancer-related death for gastric cancer because of the low early diagnosis rate (11). The liver is the most common and important metastatic organ, thanks to the abundant blood circulation between two organs. Patients with GCLM have significantly poorer prognosis, and the role of surgical treatment in such patients has always been highly controversial. In this study, we analyzed the survival impact of PTR treatment on the GCLM patients and ulteriorly constructed and validated a nomogram predicting the prognosis of surgical patients.

In theory, the resection of primary lesion for advanced gastric cancer could prolong survival time of patients by diminishing tumor burden and relieving immunosuppression caused by tumor. Nevertheless, some studies have shown diametrically opposite results and proposed contrary suggestions. NCCN clinical practice guidelines and certain clinical studies advocate palliative chemotherapy rather than surgical treatment of primary tumor for stage IV patients (2, 12). According to the follow-up result of REGATTA trial, a phase 3 randomized controlled trial, gastrectomy plus chemotherapy (S-1 + cisplatin) did not improve the overall survival of stage IV patients when compared to chemotherapy alone. By contrast, some retrospective research results suggest that some patients who meet certain requirements could benefit from surgery (4, 13–15). Early N stage and less number of liver metastases in GCLM patients were associated with a better overall survival (5, 16). Similarly, our study showed that the overall survival of GCLM patients receiving PTR is significantly better than that of patients without PTR. One of the reasons underlying this difference may be derived from the dissimilar clinical characteristics of selected patients. Most of the patients with stage IV gastric cancer in our study or in the above retrospective research only have liver metastasis, while stage IV patients with a single non-curable factor in the REGATTA trial included patients with single peritoneal metastasis or para-aortic lymph node metastasis (12).

Tumor features, such as grade of differentiation and TNM stage, have been confirmed by numerous articles to be closely related to the prognosis of GC patients (17–19). Even for GCLM patients undergoing surgery, these traditional clinical pathological features still have important prognostic significance (4, 14, 20, 21). Differentiation grade is linked to the heterogeneity and aggressiveness of tumor cells. As reported previously by some papers, there was a strong correlation between pathological differentiation feature and overall survival of GCLM patients. Patients with poorly differentiated adenocarcinoma had a worse survival (15, 22). More than that, the recent studies on GCLM patients undergoing surgery described that patients with advanced T stage (T4) could not benefit from surgery (14, 20). For instance, the result of a multicenter study including 256 patients indicated that serosal invasion is an independent predictor of poor prognosis of GCLM patients receiving gastrectomy and hepatic resection (20). In addition, several studies have concluded that GCLM patients with non-hepatic distant metastases had a short survival time and thus recommended a non-surgical treatment for those patients (21, 23). In line with the above-mentioned studies, the further investigation of the prognostic factors of GCLM patients receiving PTR in our study revealed a similar result. The poor differentiation (Grade III&IV), T4 stage, and concurrence of non-hepatic distant metastasis were key factors influencing the prognosis of patients. Unexpectedly, multivariate Cox analysis showed that N stage and histological type did not affect the survival of GCLM patients, which is contrary to the conclusions reported by earlier studies (5, 16, 24). As reported in certain literature, “Signet-ring” cells carcinomas, as extremely aggressive histological forms, is characterized by a poor survival (24).

In addition, our results showed that, as expected, perioperative adjuvant therapy, including chemotherapy, was also a significant prognostic factor for GCLM undergoing PTR. Currently, the combination of platinum and fluoropyrimidines is the first-line chemotherapy for advanced gastric cancer, which can improve the survival and quality of life of patients with locally advanced unresectable or metastatic gastric cancer (3). Preoperative neoadjuvant chemotherapy is believed to reduce the tumor size, increase the possibility of surgical resection, and improve survival (21, 25, 26). As reported by several study, perioperative chemotherapy is an important factor affecting the prognosis of GCLM patients who underwent surgical treatment (21, 27). The case in point is the AIO-FLOT3 clinical trial, a prospective Phase II clinical study, which suggested that advanced gastric cancer with localized distant metastasis could benefit from conversion surgery after preoperative chemotherapy (26).

Taken together, except adjuvant therapies, the overview of tumor including differentiation, T stage, and distant metastases can be evaluated before surgery via comprehensive inspection. Accordingly, comprehensive nomogram consists of four critical factors was developed and had the potential to assist as a basis for treatment decisions preoperatively. Similar studies, based on SEER database, had also been conducted to explore prognostic factors of GCLM patients or discuss the survival benefit of PTR for GCLM patients (15, 18). However, our research not only proved that patients with GCLM can benefit from PTR but also established nomogram for surgical patients, which the unfinished work of the former. More importantly, the nomogram has been confirmed to be effective and robust by external cohort from east Asian population, which proved that the nomogram has good generality and flexibility. Meanwhile, we identified a high-risk subgroup in GCLM patients who could not benefit from PTR, on the basis of this nomogram.

In the era of personalized and precise treatment, various molecular subtypes and clinical prediction models for GC patients have been developed one after another (28–31). Although traditional AJCC-TNM staging system still plays an important role in treatment choice, it has a lot of significant limitations for gastric cancer subgroup patients, such as GCLM patients. In this paper, we also provide a validated nomogram to assist clinicians in preoperative decision-making and improve patient prognosis. Yet, our work also has certain shortcomings. First, this is a retrospective study, and thus, prospective research with a large-scale sample is needed to confirm our discovery. Second, the information acquired from SEER database is insufficient. The specific procedure of surgery and chemotherapy regimen has not been described. It is unclear whether surgery on the metastatic lesion is performed in SEER cohort. Third, it should be noted that T stage and distant metastasis of certain organs, such as peritoneal metastasis, were difficult to diagnose accurately before surgery, which might limit the use of the model and nomogram. Furthermore, the major type of non-hepatic distant metastases in external validation was peritoneal metastasis, which differed greatly from SEER cohort, although it also reflected the flexibility of the nomogram to some extent.

## 5 Conclusions

We created and validated a prognostic nomogram for GCLM patients with PTR using the training set from SEER database and external set from Asian population. In addition, although PTR could prolong the survival of entire GCLM patients, patients in the high-risk subgroup identified by our nomogram might not benefit from surgery. In brief, our work has provided a special insight for personalized surgical treatment of GCLM patients.

## Data Availability

The raw data supporting the conclusions of this article will be made available by the authors, without undue reservation.
