# Supplementary material for: Development and validation of a nomogram to predict the survival and estimate surgical benefits for gastric cancer with liver metastasis receiving primary tumor resection
Source: Front Oncol. 2024 Nov 8;14:1418548. doi: 10.3389/fonc.2024.1418548 (PMC11581971; doi:10.3389/fonc.2024.1418548)
Supplement: Supplementary file 1 [file DataSheet1.pdf]

Supplementary Materials

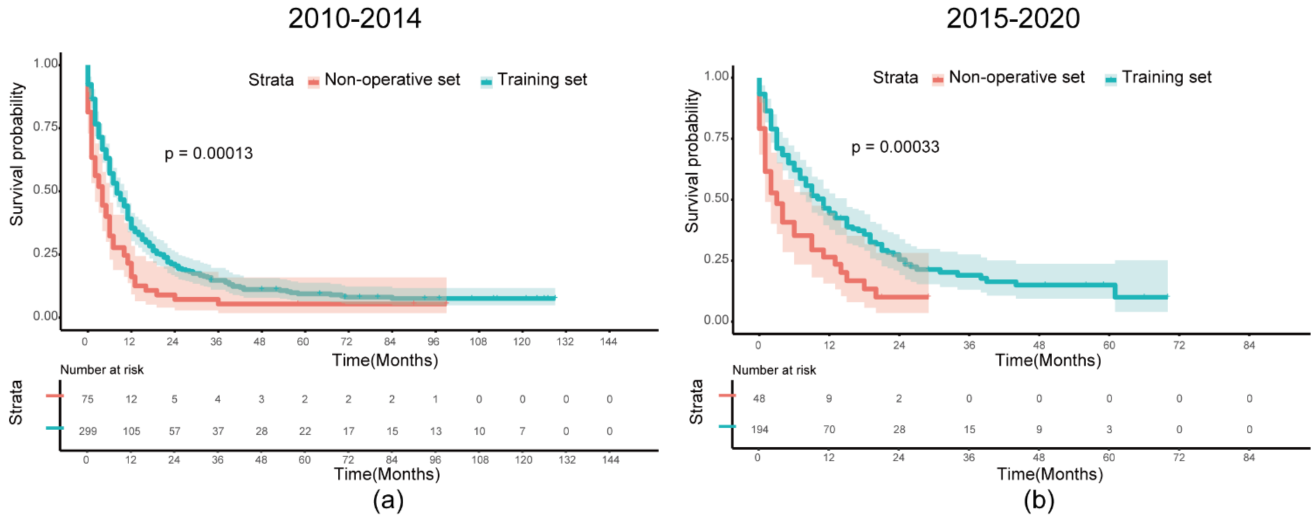

Supplementary Figure 1. Kaplan–Meier survival curves for overall survival of GCLM registered to SEER cohort between 2010-2014 (a) (Log-rank test, p=0.0001) or between 2015-2020 (b) (Log-rank test, p=0.0003) according to the implementation of PTR.

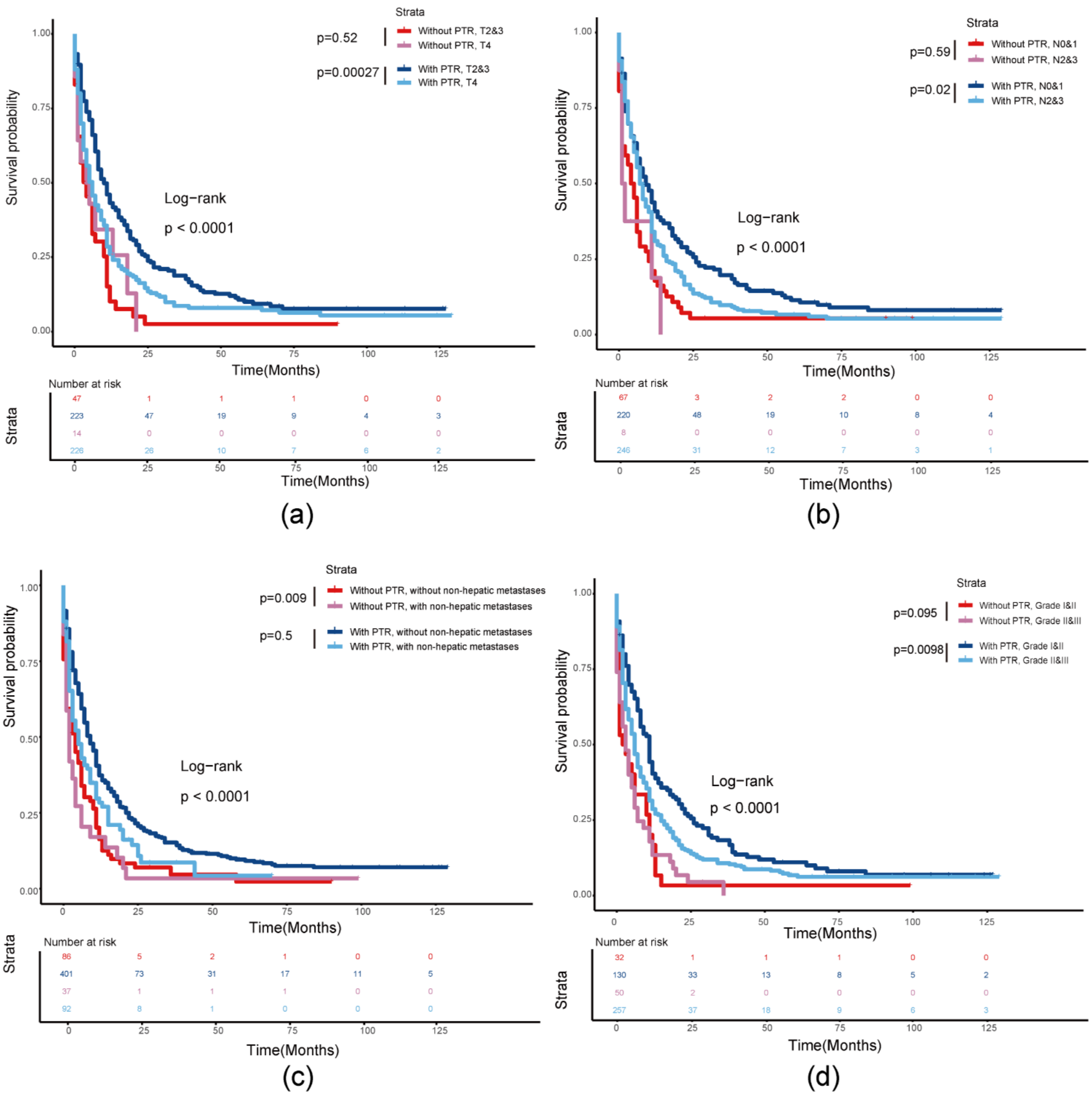

Supplementary Figure 2. Kaplan–Meier survival analysis for GCLM patients in SEER cohort further stratified according to T stage (a), N stage(b), non-hepatic metastases (c) and differentiation grade(d) within non-operative set (without PTR) and training set (with PTR).
